# An online population-representative longitudinal cognitive dataset from the Understanding America Study

**DOI:** 10.1038/s41597-026-07050-4

**Published:** 2026-03-17

**Authors:** Margaret Gatz, Jill E. Darling, Stefan Schneider, Ying Liu, Deborah Finkel, Bart Orriens, Raymond Hernandez, Tania Gutsche, Yadira Garcia, Arie Kapteyn

**Affiliations:** 1 Dornsife Center for Economic & Social Research, University of Southern California, Los Angeles, CA USA; 2 Department of Psychology, University of Southern California, Los Angeles, CA USA; 3 Leonard Davis School of Gerontology, University of Southern California, Los Angeles, CA USA; 4 Institute for Gerontology, Jönköping University, Jönköping, Sweden

**Keywords:** Dementia, Cognitive ageing, Epidemiology, Working memory

## Abstract

Cognitive epidemiology explores cognitive ability as a risk factor for various health and disease-related outcomes. To support research in this area, the Understanding America Study (UAS)–a nationally representative online probability sample–has developed and collected longitudinal assessments of multiple cognitive domains (e.g., fluid intelligence, executive function, processing speed, verbal episodic memory). These assessments have been compiled into the Cognitive Comprehensive File (CogCF), a publicly available resource of cognitive test data for over 21,000 adults aged 18 years and older. While valuable on its own, the CogCF may also be linked to an extensive range of other publicly available data collected in the UAS, including the complete Health and Retirement Study survey instrument and additional surveys on topics such as job history, personality, financial and psychological wellbeing, healthcare usage, and physical and mental health. These data enable researchers to examine critical questions such as the associations of cognitive ability with everyday life outcomes and factors associated with cognitive changes over the life course.

## Background & Summary

Cognitive epidemiology is the examination of cognitive functioning as a risk factor for various health and disease related outcomes in adults^1^. For instance, cognitive epidemiologists may examine how cognitive function relates to preventive health behaviors^1^ or health relevant outcomes such as the ability to perform instrumental activities of daily living (e.g., grocery shopping and paying bills^2^) or mortality due to chronic conditions such as coronary heart disease^3^. Several domains of cognitive functioning exist, each with its own implications for health and functioning^4^. For instance, cognitive language skills are particularly relevant to engagement in social activities, while episodic memory is key to recollection of everyday experiences^4^. Thus, comprehensive epidemiological investigations require assessing multiple cognitive domains.

Historically, panel studies that include cognitive assessment modules have employed in-person cognitive testing, conducted during home visits or by bringing participants into a laboratory or clinic. Telephone testing has been used to reach more participants. Computer-based tests were developed for lab or clinic use, increasing standardization of test protocols and precision of measurement of timed tests. More recently, self-administered computer-based tests are increasingly deployed. Proponents of online cognitive testing have highlighted its greater cost-effectiveness and broader accessibility^5^, with evidence suggesting that this can yield scores comparable to those from lab-based testing^5,6^. To provide high quality data, online cognitive testing requires careful development and implementation. This includes making sure that scores are comparable across device types (e.g., computer and smartphone), designing clear instructions so they can be understood without interviewer support, creating user-friendly interfaces for individuals with low technological proficiency, and taking steps such as adding on-screen prompts to ensure that participants complete surveys with minimal distractions^7^. Some longstanding panel studies, particularly those focused on aging, have begun shifting to online data collection that has also entailed online cognitive testing, including the National Social Life, Health, and Aging Project^8^, the Trøndelag Health study^9^, and the Health and Retirement Study^10^.

The Cognitive Comprehensive File (CogCF) is a longitudinal database developed for the Understanding America Study (UAS), aiming to encompass a broad range of cognitive functioning of U.S. adults with data collection always fully online. The UAS is a nationally representative longitudinal online survey panel of U.S. residents aged 18 and older, which was first launched in 2014 yet is ongoing and continues to grow^11^. Given the centrality of online administration to its structure, the UAS assembled a cognition workgroup with expertise in cognitive functioning, psychometrics, and web development to prepare, adapt, and compile cognitive tests specifically for online administration. Test development involves an iterative process of pilot testing, participant feedback, and refinement^7^. Because the UAS incorporates core survey questions from the Health and Retirement Study (HRS), the cognition workgroup initially developed and implemented online versions of in-person tests from the HRS, including the modified Telephone Interview of Cognitive Status (TICS)^12^ and Woodcock-Johnson tests of Cognitive Abilities^13^ that had been adapted for the HRS^14^. Other components of the CogCF reflect the emphasis of the UAS on issues of economics and personal finance. To these were added assessments of cognitive domains that are sensitive to both typical cognitive aging and pathological decline^15^, including measures of executive functioning and perceptual speed^7^.

In addition to the cognitive domains covered by the CogCF database, CogCF data are directly linkable, via an anonymized study ID number, to a broader range of other UAS datasets on the same participants, a critical advantage necessary for studying cognitive epidemiology. Frequently linked UAS datasets include: mental and physical health, financial literacy, neighborhood quality, retirement choices, just to name a few. Studies have used such linked data to investigate meaningful research questions such as the relationships between COVID-19 infections and cognitive functioning^16^, objective and subjective financial literacy^17^, as well as links between numeracy and medical outcomes^18^, and the ability to value annuities^19^.

## Methods

Detailed methods describing the UAS study generally are previously available from the UAS website at https://uasdata.usc.edu/page/Documentation^20^ and from the UAS protocol paper,^11^ while comprehensive information on the CogCF file can be found in the “UAS Cognitive Comprehensive File Data Description” document (https://uasdata.usc.edu/page/Cognitive+Comprehensive+File)^21^. Methods for both are summarized below.

The UAS is a nationally representative probability sample designed to investigate a wide array of social, behavioral, and health-related topics^11^. Establishment of the UAS was in part driven by the recognition that while national surveys were traditionally conducted in person, online survey administration would play an increasingly important role moving forward. Established in 2014, the UAS is housed at the Center for Economic and Social Research (CESR) at the University of Southern California. Today, it serves as an open research platform with a panel of U.S. residents who are actively participating, offering an extensive infrastructure for longitudinal studies. Recruitment into the UAS is ongoing.

As long as UAS participants remain in the panel after enrollment, they complete core surveys – including the cognitive tests for the CogCF database – every two years, along with several non-core surveys^11^. Informed consent was obtained from all individual participants included in the UAS prior to their taking part, and the study received ethical approval from the Biomedical Research Alliance of New York (BRANY) Institutional Review Board (22-0301044). On average, UAS participants complete surveys once or twice a month, with each survey lasting no more than 30 minutes and compensation proportional to survey length. Participants are invited to complete online surveys (including cognitive assessments) using their own devices (i.e., computers, smartphones, or tablets) or using UAS-provided tablets if they report not having a device. The survey platform is designed to ensure a standardized and user-friendly experience, featuring clear instructions, time limits where applicable, and automated data recording. Because participants join the UAS at different timepoints, the timing of core surveys is relative to their enrollment date. Participants are invited to complete a set of core surveys upon joining, and then to repeat these core surveys approximately 2 years after they completed the prior wave. As a result of this tailored enrollment, the amount and timing of data collected vary across participants.

Administering cognitive tests biennially allows for repeated assessments over time and is consistent with the frequency at which other core information is collected. This schedule follows other population surveys, such as the Panel Study of Income Dynamics and the Health and Retirement Study^22^. Biennial testing is likely to minimize the effect of practice effects, while also striking a balance between participant burden and collecting data with a frequency that is granular enough to examine longitudinal change. The tests are administered in separate surveys to prevent fatigue effects.

The CogCF file is continuously growing as more waves of cognitive tests continue to be administered, but information in the sections that follow are based on CogCF data downloaded from the UAS website on January 15, 2026, which can be accessed at the following link after a UAS account has been created and while logged in on the UAS website: https://uasdata.usc.edu/index.php?r=eNpLtDKyqi62MrFSKkhMT1WyLrYyNAeyS5NyMpP1UhJLEvUSU1Ly80ASQDWJKZkpUKahoZmRknUtXDB_yBMQ. Because of the large amount of information on participant characteristics recorded at each cognitive testing event, participant demographic information is stored in a separate file. The version used in this paper can also be accessed in the link provided.

### Participants

The UAS employs Address-Based Sampling (ABS), ensuring that all U.S. residents aged 18 and older have a non-zero probability of selection^11^. Participants are first reached by regular mail, and those without a digital device are provided with internet-enabled tablets, mitigating digital divide concerns and enhancing sample representativeness. UAS surveys are conducted in English and Spanish. Individual survey response rates have been found to be around 75%^11^. To maintain a nationally representative sample, different groups (i.e., African American, Asian, Hispanic, and rural participants) are oversampled to ensure that they are adequately included in the study. Furthermore, to retain participants in the study, regular reminders are sent for uncompleted surveys and there is a protocol for following up with them to encourage re-engagement for up to approximately a year after their last survey response. Table 1 describes the demographic characteristics of all CogCF participants when they completed their first cognitive survey, typically the financial literacy and numeracy tests. Table 2 describes the baseline demographic characteristics of CogCF participants who completed their first cognitive survey when they were age 50 or older.

**Table 1:** Demographic characteristics of CogCF participants at the completion of their first cognitive survey (N = 21,526, as of January, 2026).

	Count	Percent
Age		
18–24	1879	8.8
25–34	4197	19.6
35–49	6103	28.4
50–64	5678	26.5
65 or older	3601	16.8
Sex		
Female	12797	59.5
Male	8714	40.5
Race		
White Only	15315	71.7
Black Only	2541	11.9
Asian Only	1582	7.4
Mixed	1227	5.7
American Indian or Alaska Native Only	526	2.5
Hawaiian/Pacific Islander Only	183	0.9
Hispanic/Latino		
Hispanic	3706	17.2
Non-Hispanic	17803	82.8
Language		
English	21154	98.3
Spanish	369	1.7
Education		
High school or lower	4870	22.6
Some college no degree	4752	22.1
Associate’s degree	2779	12.9
Bachelor’s degree	5267	24.5
Master’s degree or higher	3836	17.8
Household Income		
<$25,000	4625	21.6
$25,000-$49,999	4506	21
$50,000-$74,999	3911	18.3
$75,000 to $99,999	2701	12.6
≥$100,000	5670	26.5
Completion Year of First Cognitive Survey		
2014	1694	7.9
2015	703	3.3
2016	4075	18.9
2017	533	2.5
2018	1424	6.6
2019	2151	10
2020	1639	7.6
2021	1121	5.2
2022	1497	7
2023	4085	19
2024	2236	10.4
2025	356	1.7
2026	9	0

**Table 2:** Baseline demographic characteristics of CogCF participants who completed their first cognitive survey at age 50 or older (N = 9,275 as of January, 2026).

	Count	Percent
Age		
50–64	5678	61.2
65–74	2615	28.2
75–84	854	9.2
85 or older	132	1.4
Sex		
Female	5055	54.5
Male	4223	45.5
Race		
White Only	7127	77.1
Black Only	1050	11.4
Asian Only	485	5.2
Mixed	394	4.3
American Indian or Alaska Native Only	150	1.6
Hawaiian/Pacific Islander Only	42	0.5
Hispanic/Latino		
Hispanic	802	8.6
Non-Hispanic	8475	91.4
Language		
English	9174	98.9
Spanish	105	1.1
Education		
High school or lower	1978	21.3
Some college no degree	2107	22.7
Associate’s degree	1257	13.6
Bachelor’s degree	2113	22.8
Master’s degree or higher	1821	19.6
Household Income		
<$25,000	1783	19.3
$25,000-$49,999	1986	21.5
$50,000-$74,999	1752	19
$75,000 to $99,999	1204	13.1
≥$100,000	2494	27.1
Completion Year of First Cognitive Survey		
2014	813	8.8
2015	227	2.4
2016	2032	21.9
2017	218	2.3
2018	651	7
2019	661	7.1
2020	700	7.5
2021	487	5.2
2022	650	7
2023	1727	18.6
2024	1013	10.9
2025	100	1.1

### Contents of the CogCF Dataset

For each participant, the CogCF dataset includes demographic and household characteristics, scores on each test taken and the dates of the tests, and other cognition relevant measures and their corresponding dates. Variable names for cognitives scores, dates, and participant characteristics are preceded by a letter indicating the particular study and number indicating the wave of the study. Because the UAS sample has expanded and because cognitive tests have been added over time, the maximum number of biennial waves a participant has been eligible to complete at any given time is different for each test, and dependent on the date that the participant joined the UAS panel. As of January 2026, six waves have been administered for one test (Serial 7s), five waves for five of the tests (Financial Literacy, Numeracy, Number Series, Analogies, and Picture Vocabulary), and three waves for five of the tests (Stop & Go Switch Task (SGST), Figure Identification, Word Recall, Box Clicking, and Typing Speed). Most tests are presented on separate dates as stand-alone surveys. However, Serial 7s, Word Recall, Box Clicking, Typing Speed, and Self-Reported Memory are part of the same core survey (i.e., completed at the same time). Also, Financial Literacy and Numeracy are in the same core survey. Available cognition relevant measures that are not cognitive tests include two measures of subjective cognition–Self Reported Memory (part of the core survey as Word Recall) and Perceived Cognitive Function–and a calculated score indicating the probability of cognitive impairment (for those aged 50 + only).

**Fig. 1:**
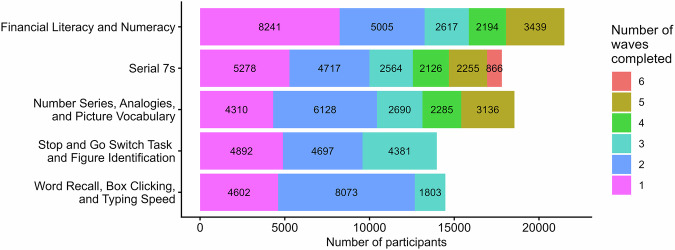
Sample size by counts of completed waves for each cognitive test (as of January 2026).

Figure 1 describes the sample size distribution for each cognitive test and provides counts of individual waves, whether sequential or non-contiguous, completed as of January 15, 2026.

**Fig. 2:**
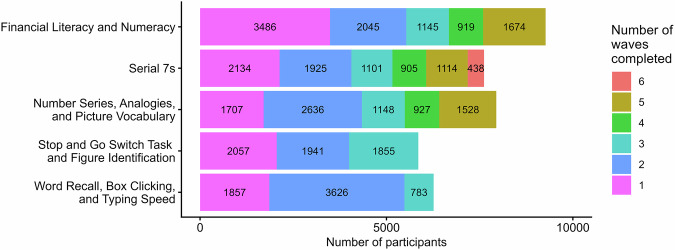
Sample size by counts of completed waves for each cognitive test, only for participants who were age 50 or older when completing their first cognitive survey.

Figure 2 describes the sample size distribution for each cognitive test and provides counts of individual waves, only for participants who were age 50 or older when completing their first cognitive survey.

#### Cognitive tests

Each test is administered using a standardized protocol to ensure consistency across administrations. Below we described them by launch year, and list the variable names (with a letter prefix for the study and number prefix for the wave, like “p1”). Note that different versions of the scores are sometimes available, and they are described in greater detail in the CogCF documentation^21^.

- Financial Literacy (5 waves, first administered in 2014): Asks 14 questions about **financial literacy**, with higher scores indicating greater literacy^23,24^. Questions asked include ones addressing stock market knowledge and compound interest. The corresponding variable names in the CogCF file are “p1finlitscore”, “p2finlitscore”, “p3finlitscore”, “p4finlitscore”, and “p5finlitscore.”
- Numeracy (5 waves, first administered in 2014): Participants complete 8 questions testing **numeracy** that are scored with Item Response Theory (IRT) based scale scores^25^. Questions asked include ones addressing probability and monetary spending relevant computations. The corresponding variable names are “p1cog”, “p2cog”, “p3cog”, “p4cog”, and “p5cog.”

- Serial 7s (6 waves, first administered in 2014): Assesses **sustained attention** and **working memory**^26^. Participants are asked sequentially to subtract seven from 100 five times. One point is awarded for each correct subtraction, for a maximum score of 5 and minimum of 0. The corresponding variable names are “r1serialseven”, “r2serialseven”, “r3serialseven”, “r4serialseven”, “r5serialseven”, and “r6serialseven.”

- Number Series (5 waves, first administered in 2016): Assesses **fluid intelligence** by presenting participants with a series of four numbers with one number missing, and asking them to identify the missing number based on the pattern^14^. IRT-based normed scores are derived using a two-parameter logistic IRT model. Two parallel forms are administered in alternating order to reduce practice effects. The corresponding variable names are “n1num_cog”, “n2num_cog”, “n3num_cog”, “n4num_cog”, and “n5num_cog.”
- Verbal Analogies (5 waves, first administered in 2016): Assesses **fluid intelligence** by prompting participants to identify relationships between word pairs^14^. IRT-based normed scores for two alternating parallel forms are calculated using a two-parameter logistic IRT model. The corresponding variable names are “a1vana_cog”, “a2vana_cog”, “a3vana_cog”, “a4vana_cog”, and “a5vana_cog.”
- Picture Vocabulary (5 waves, first administered in 2016): Tests **word knowledge** by presenting participants with a series of pictures and asking them to type the names of each one^13^. IRT-based normed scores for two alternating parallel forms are calculated using a two-parameter logistic IRT model. The corresponding variable names are “v1pvoc_cog”, “v2pvoc_cog”, “v3pvoc_cog”, “v4pvoc_cog”, and “v5pvoc_cog.”

- Stop and Go Switch Task (3 waves, first administered in 2020): Assesses executive functions by measuring median response times to a congruent and a non-congruent rule and then switching between between congruent and incongruent response rules each time the participant is cued to switch^27,28^. The response times capture **choice reaction time** (“g1nb_score”, “g2nb_score”, and “g3nb_score”), **response inhibition** (“g1rb_score”, “g2rb_score”, and “g3rb_score”), **task switching** (“g1sw_score”, “g2sw_score”, and “g3sw_score”), and **sustained attention/response consistency** (“g1nsw_score”, “g2nsw_score”, and “g3nsw_score”), respectively. Faster response times (i.e., a lower value) indicate greater ability. Note that these scores were set to missing if less than 70% of trials were correct, suggesting likely careless responding for the event, but another version of these scores without this percentage correct requirement is also available (e.g., “g1nb_score_all”).
- Figure Identification Test (3 waves, first administered in 2020): Assesses **perceptual speed** by presenting participants with one figure on top of 5 horizontally aligned similar figures^29^. Participants are prompted to select one figure from the 5 that exactly matches the one top figure as quickly as possible while being accurate. The test uses two alternating parallel forms to reduce practice effects. The corresponding variable names are “u1figID_score”, “u2figID_score”, and “u3figID_score.” Note that these scores were set to missing if less than 70% of trials were correct, suggesting likely careless responding for the event, but another version of these scores without this percentage correct requirement is also available (e.g., “u1figID_score_all”).
- Word Recall (3 waves, first administered in 2021): Tests **verbal episodic memory** with immediate and delayed word recall tasks^12,30^. Participants are presented with a list of 10 words and asked to recall them immediately and after a delay of several minutes. The score is the number of words correctly recalled. To minimize practice effects, four alternative word lists are used. The corresponding variable names for immediate recall are “r4recall1_score”, “r5recall1_score”, and “r6recall1_score”, while for delayed recall they are r4recall2_score”, “r5recall2_score”, and “r6recall2_score”,.
- Brief Box Clicking Test (3 waves, first administered in 2021): Assesses **processing speed** and **visual motor integration**^31^. Participants are presented with four stationary boxes arranged in a square and are asked to click (or tap) each box as quickly as they can. The corresponding variable names are “r4box_click_score”, “r5box_click_score”, and “r6box_click_score.” Faster response times (i.e., a lower value) indicate greater ability. Response times were log-transformed to normalize their distribution. Box clicking times above the 99th percentile (90 seconds) were set to missing as they were considered outliers, consistent with prior treatment of response time data.
- Typing Speed (3 waves, first administered in 2021): Captures **typing speed/performance**^32^. Participants instructed to “type the sentence below as quickly as you can” in English [“The quick brown fox jumps over the lazy dog”] or Spanish [“Cada vez que trabajo, Felix me paga un whisky.”] Typing speed is operationalized as “adjusted typing speed”, a metric that is the product between typing speed and accuracy, thereby taking both into account. The corresponding variable names are “r4lawpm”, “r5lawpm”, and “r6lawpm.” Typing speed for observations with less than 50% accuracy were set to missing for being over erroneous.

#### Other cognition relevant measures

The CogCF dataset also includes several derived and self-reported variables that are relevant to cognition but are not cognitive tests, described below.

- Activities of Daily Living (6 waves, first administered in 2015): Self-reported **difficulties with activities of daily living (ADLs) and/or instrumental activities of daily living (IADLs)** are captured with the HRS item: “Because of a health or memory problem do you have any difficulty with (ADL/IADL)? ADLs addressed are dressing, eating, walking, getting in/out of bed, using the toilet, and bathing. IADLs addressed are meal preparation, making a phone call, shopping for groceries, taking medications, house/yard work, and money management.
- Self-Reported Memory (6 waves, first administered in 2015): **Present memory status** is assessed with the item “First, how would you rate your memory at the present time?” (1 excellent to 5 poor). Perceived **change in memory status** is assessed with the item “Compared to two years ago, would you say your memory is better now, about the same, or worse than it was then?” Response options are “better”, “stayed the same”, or “worse”.
- Probability of Cognitive Impairment (PCI) Score (3 waves, first computed in 2021)**:** Captures the **probability of cognitive impairment** with a composite measure incorporating scores from the word recall (i.e., immediate and delayed recall) and serial 7s tasks^33^. A version is also available that incorporates functional difficulties (ADL/IADL). The PCI score is derived through a latent-variable modeling approach validated against the Langa-Weir criteria used in the HRS to classify individuals into categories of normal cognition, cognitive impairment but no dementia, and dementia^34^.

- Perceived Cognitive Function (1 wave, first administered in 2024): **Subjective cognitive abilities and concerns about cognitive difficulties in daily life** are assessed with the PROMIS® Cognitive Function measure^35^. Participants complete a 4-item Computerized Adaptive Test (CAT), which dynamically selects items from the calibrated PROMIS Cognitive Function item bank based on each participant’s prior responses. This adaptive approach optimizes measurement precision while minimizing respondent burden^36^. Higher scores reflect better perceived cognitive function.

Data from the CogCF dataset described here are publicly available through the UAS data portal (https://uasdata.usc.edu/index.php?r=eNpLtDKyqi62MrFSKkhMT1WyLrYyNAeyS5NyMpP1UhJLEvUSU1Ly80ASQDWJKZkpUKahoZmRknUtXDB_yBMQ) and downloadable while logged in on the UAS website. Cognitive test data are categorized as Tier 1 data, meaning they exclude direct or indirect identifiers and are readily available for download after prospective users apply for a data user account on the UAS data site and sign a Data User Agreement (DUA). CogCF relevant files available for download include the main CogCF file with all the main cognitive variables, a file with wave-specific demographic information associated with the cognitive testing data^37^, and a data description^21^. Users would benefit from referring first to this data description, as it provides an in-depth explanation of the naming conventions used for CogCF variables and provides more extensive background on scoring procedures used for each cognitive test.

## Technical Validation

Cognitive tests are implemented online in an iterative process of pilot testing, empirical validation, and test refinement^7^, in addition to collecting participant feedback, particularly to increase participants’ response rate and engagement^38^. Tests that are well-established in cognitive research were selected and adapted for online administration, initially the modified TICS (as part of the core HRS^12^) and tests adapted by CogUSA^39^ for online administration. For tests that require substantial adaptation or are specifically developed with the UAS, we have conducted validity testing typically in the first wave of their data collection. In addition to studies that examine score reliability^15^, test-retest stability (across a two-year period given the biennual test administration)^31,32^, and age gradient both across different individuals^7,31,32^ and across multiple measures of the same individuals^15^, we have designed validation studies to address specific research questions that could potentially impact the psychometric properties of individual cognitive measures. For example, because the Stop and Go Switch task and Figure Identification test both utilize visual cues and rely on response time for scoring and thus could be sensitive to platform differences, we compared test performance using a keyboard- and mouse-based device versus a touch screen-based device and found little difference^7^. In development of Box Clicking^31^ and Typing Speed^32^ scores, we found that the two scores moderately correlated with other assessments of visual-perceptual speed as well as functional capacity and motor coordination. To develop the Probability of Cognitive Impairment metric^33^, we crosswalked the well-established phone-based modified TICS score to the adapted web-based counterpart and further showed that individuals with higher score in the metric were more likely to report having received a dementia diagnosis from a physician. Additional details on test-specific psychometric properties and routine quality control can be found in the CogCF data documentation^12^.

We have also examined the extent to which environmental influences could potentially impact cognitive performance, including the location of participants, other activities they perform simultaneously, and whether they were interrupted while completing the test. Results indicated that being interrupted could be a key confounder^7^. Thus, a self-reported distraction question is added at the end of each cognitive test, with these data available in the individual survey data file. Interested data users could use these data for further data cleaning or robustness check.

For tests where scores were dependent on response speed, we examined the effect of type of device used to complete the test (i.e., computer versus smartphone)^7,31,32^. Information on type of device used is available for individual UAS surveys through their associated “platform data” file on the UAS website^40^.

Here we additionally provide descriptive statistics and associations with language, age, and education for the eleven cognitive tests. Only data from the first time participants completed each cognitive test were used to remove the influence of practice effects and to avoid giving greater weight in results to participants who completed tests multiple times. The effect of language (i.e., English versus Spanish) was examined by regressing cognitive test scores on a binary variable (1 if participants requested surveys in English and 0 if Spanish) in bivariate models. Pearson correlations were computed between cognitive test scores and age. Model predicted means and standard errors were computed by gradients of age and education. Finally, all available data were used to compute the intraclass correlation coefficient (ICC) for each cognitive test, a reliability metric that in this context takes on the meaning of test-retest stability across approximately two years, or the proportion of variance in tests that is due to stable between-person differences as opposed to within-person fluctuations across waves. ICCs can be interpreted using commonly applied benchmarks: poor (0–.19), fair (.20–.39), moderate (.40–.59), substantial (.60–.79), and almost perfect (.80–1.0) stability^41^.

Table 3 shows descriptive statistics, associations with language and age, and ICCs for all the cognitive tests. With regards to language, people that completed surveys in English had higher scores on all the cognitive tests as compared to those that completed it in Spanish, consistent with prior work^42^. Note however that, as indicated in Table 1, the vast majority of participants (98%) opted to complete surveys in English. No language estimates were listed for the Verbal Analogies and Picture Vocabulary tests because too few participants completed the tests in Spanish for the IRT based scores to be reliably computed^21^. As expected, greater age was correlated with better performance on the crystallized intelligence oriented measures, Financial Literacy and Picture Vocabulary. For the other tests, with the exception of Serial 7s scores, better performance was associated with lower age. The average ICC across all cognitive tests was 0.60, suggesting moderate stability across time for most of the test scores.

**Table 3:** Descriptive statistics and associations with language and age based on scores from the first completion of each cognitive test.

	Mean	SD	Range	Language effect B (p)	Corr. with age r (p)	ICC
Financial Literacy	9.26	3.25	0-14	2.84 (p < 0.001)	0.26 (p < 0.001)	0.76
Numeracy	51.03	9.06	33.54–70.45	5.98 (p < 0.001)	−0.03 (p < 0.001)	0.77
Serial 7s	4.4	1.16	0–5	0.16 (p = 0.025)	0.02 (p = 0.042)	0.38
Number Series	50.83	9.39	13.58–65.34	6.22 (p < 0.001)	−0.02 (p = 0.028)	0.75
Verbal Analogies	50.21	8.99	13.14–69.67	NA	−0.03 (p < 0.001)	0.69
Picture Vocabulary	49.3	9.16	12.53–74.36	NA	0.42 (p < 0.001)	0.76
Stop & Go Switch Task choice reaction time^a^	1.03	0.64	0.25–30	−0.47 (p < 0.001)	0.34 (p < 0.001)	0.36
SGST response inhibition^a^	1.1	0.67	0.1–30.65	−0.11 (p = 0.081)	0.31 (p < 0.001)	0.53
SGST sustained attention, response consistency^a^	0.92	0.33	0.38–6.41	−0.14 (p < 0.001)	0.46 (p < 0.001)	0.5
SGST task switching^a^	1.5	0.69	0.21–12.87	−0.47 (p < 0.001)	0.33 (p < 0.001)	0.49
Figure Identification	18.13	5.96	2–30	3.07 (p < 0.001)	−0.52 (p < 0.001)	0.76
Immediate Word Recall	5.03	2.32	0–10	0.86 (p < 0.001)	−0.03 (p < 0.001)	0.51
Delayed Word Recall	3.88	2.53	0–10	0.92 (p < 0.001)	−0.03 (p < 0.001)	0.54
Box Clicking time^a^	2.52	0.54	−0.09–4.5	−0.26 (p < 0.001)	0.36 (p < 0.001)	0.52
Typing Speed	3.47	0.6	1.15–5.2	0.40 (p < 0.001)	−0.49 (p < 0.001)	0.71

Computation of the intraclass correlation coefficient (ICC) involved consideration of all cognitive testing scores, not just the first completion.

^a^For these cognitive tests, unlike the other scores, higher values are indicative of worse functioning.

Table 4 depicts predicted means by gradients of age and education. The mean test scores by age group are consistent with the correlations from Table 3. Across all cognitive tests, greater levels of education were generally associated with higher mean scores, consistent with prior work^43^.

**Table 4:** Mean scores from the first completion of each cognitive test, by age and education based groups.

	Age 18–24, μ (SE)	Age 25–34, μ (SE)	Age 35–49, μ (SE)	Age 50–64, μ (SE)	Age 65 + , μ (SE)	HS or lower, μ (SE)	Some college, μ (SE)	Assoc. degree, μ (SE)	BS/ BA, μ (SE)	MS/ MA or higher, μ (SE)
Financial Literacy	7.42 (0.07)	8.46 (0.05)	9.15 (0.04)	9.81 (0.04)	10.44 (0.05)	7.02 (0.04)	8.60 (0.04)	8.95 (0.05)	10.62 (0.04)	11.24 (0.05)
Numeracy	50.75 (0.21)	51.65 (0.14)	51.18 (0.12)	50.60 (0.12)	50.90 (0.15)	45.74 (0.12)	49.29 (0.12)	49.40 (0.16)	54.66 (0.11)	56.11 (0.13)
Serial 7s	4.33 (0.03)	4.37 (0.02)	4.41 (0.02)	4.42 (0.02)	4.42 (0.02)	4.03 (0.02)	4.36 (0.02)	4.39 (0.02)	4.61 (0.02)	4.64 (0.02)
Number Series	50.08 (0.24)	51.21 (0.16)	51.18 (0.13)	50.61 (0.13)	50.54 (0.17)	45.67 (0.13)	49.22 (0.13)	49.49 (0.18)	54.32 (0.13)	55.49 (0.15)
Verbal Analogies	48.99 (0.23)	50.59 (0.15)	50.84 (0.13)	50.15 (0.13)	49.42 (0.16)	45.55 (0.14)	49.09 (0.14)	49.52 (0.18)	53.08 (0.13)	53.85 (0.15)
Picture Vocabulary	42.39 (0.21)	45.07 (0.14)	48.41 (0.12)	52.07 (0.12)	54.65 (0.15)	45.26 (0.14)	48.98 (0.14)	49.06 (0.18)	51.11 (0.13)	52.33 (0.16)
Stop & Go Switch Task choice reaction time^a^	0.74 (0.02)	0.80 (0.01)	0.91 (0.01)	1.13 (0.01)	1.39 (0.01)	1.12 (0.01)	1.05 (0.01)	1.05 (0.02)	0.97 (0.01)	0.96 (0.01)
SGST response inhibition^a^	0.85 (0.02)	0.89 (0.01)	1.00 (0.01)	1.20 (0.01)	1.45 (0.01)	1.18 (0.01)	1.13 (0.01)	1.15 (0.02)	1.04 (0.01)	1.07 (0.01)
SGST sustained attention, response consistency^a^	0.73 (0.01)	0.77 (0.01)	0.85 (0.00)	0.99 (0.01)	1.17 (0.01)	0.97 (0.01)	0.95 (0.01)	0.95 (0.01)	0.88 (0.01)	0.90 (0.01)
SGST task switching^a^	1.25 (0.02)	1.28 (0.01)	1.38 (0.01)	1.57 (0.01)	1.90 (0.01)	1.62 (0.01)	1.56 (0.01)	1.54 (0.02)	1.41 (0.01)	1.42 (0.01)
Figure Identification	22.87 (0.17)	21.72 (0.10)	19.66 (0.08)	16.57 (0.08)	13.63 (0.09)	17.27 (0.11)	17.83 (0.11)	17.53 (0.14)	19.04 (0.10)	18.63 (0.11)
Immediate Word Recall	5.25 (0.08)	5.16 (0.05)	4.96 (0.04)	5.00 (0.04)	5.00 (0.04)	4.10 (0.04)	4.84 (0.04)	4.93 (0.05)	5.56 (0.04)	5.66 (0.04)
Delayed Word Recall	4.08 (0.09)	4.01 (0.05)	3.82 (0.04)	3.84 (0.04)	3.86 (0.05)	2.91 (0.04)	3.72 (0.04)	3.71 (0.06)	4.42 (0.04)	4.57 (0.05)
Box Clicking time^a^	2.19 (0.02)	2.27 (0.01)	2.43 (0.01)	2.64 (0.01)	2.77 (0.01)	2.59 (0.01)	2.56 (0.01)	2.58 (0.01)	2.46 (0.01)	2.44 (0.01)
Typing Speed	3.89 (0.02)	3.82 (0.01)	3.61 (0.01)	3.32 (0.01)	3.05 (0.01)	3.25 (0.01)	3.36 (0.01)	3.36 (0.01)	3.63 (0.01)	3.67 (0.01)

^a^For these cognitive tests, unlike the other scores, higher values are indicative of worse functioning

## Usage Notes

The CogCF file, the file with its corresponding demographic information, and many other UAS files are available after registration on the UAS website and signing a data use agreement (DUA) which is accessible at the following link: https://uasdata.usc.edu/page/Data+Agreement^44^. Registration can be completed by clicking the “Login/Register” tab of the UAS website (https://uasdata.usc.edu)^45^. The purpose of the DUA is largely to ensure that users do not make attempts to identify individuals in the UAS dataset, and do not share copies of the dataset to other individuals as everyone interested in the data should follow the registration process. After a brief review of the registration information by the UAS team, registrants will be granted access to the data.

This Data Descriptor presents a static version of the CogCF dataset (and the corresponding CogCF demographics dataset) at the UAS repository (downloaded on January 15, 2026), peer reviewed at the time of submission, and accessible at the following link after creation of a UAS account and when logged in on the UAS website: https://uasdata.usc.edu/index.php?r=eNpLtDKyqi62MrFSKkhMT1WyLrYyNAeyS5NyMpP1UhJLEvUSU1Ly80ASQDWJKZkpUKahoZmRknUtXDB_yBMQ. However, data collection is ongoing and the CogCF file is updated nightly. These further updates, created after peer review, will be available under the “UAS Cognitive comprehensive file” section of the “data products” tab, under “curated data” (https://uasdata.usc.edu/page/UAS+Cognitive+Comprehensive+File).

The documentation on the CogCF file available on the UAS website^21^ has detailed information on the cognitive tests, in addition to the variable names associated with each test. Note that the STATA version of the CogCF file has brief descriptions of the variables integrated into it.

Users will often be interested in using the CogCF file in conjunction with other UAS data files, requiring minimal data management in the form of merging datasets using an anonymized study ID number. A full description of the wide range of topics covered in the UAS is available on the UAS website and in the UAS cohort description^11^. One likely useful file is the UAS comprehensive file, which has the non-cognitive information collected from all the core surveys merged together into one file^46^. Examples of variables covered by the UAS core surveys include health history, job status, job history, healthcare coverage information, healthcare usage, income and assets, life satisfaction, neighborhood quality, social security knowledge, mental health, personality, and engagement in health behaviors. Non-core surveys further capture a wide variety of variables (e.g., political beliefs, views on education, COVID exposure, etc.) that are also accessible to UAS data users. Additionally, monthly surveys capture self-reported cognition, mental health, pain, stress, and life shocks, including recent events and natural disasters. Researchers may also access more sensitive data by applying for access to the secure UAS LINKAGE Enclave online platform, where cognitive variables can be linked to geocoded environmental variables, such as air quality and neighborhood attributes, as well as claims data for Medicare beneficiaries housed at the Centers for Medicare and Medicaid Services (CMS).

Note that the exact code required for merging the CogCF with other UAS data depends largely on the research questions of interest. In the code provided under “code availability”, syntax is provided for the simplest case where researchers wish to merge the CogCF file with other UAS files that have one row per participant (like the demographic information for the CogCF and the UAS comprehensive file). Here, merging is performed by “uasid.” Additionally, code is provided for the common task of setting the values of two variables of interest to missing if their recording dates are considered too far apart temporally (e.g., more than a year removed from one another) for the analysis of interest. For example, if researchers are interested in the association between retirement beliefs and scores on one of the cognitive tests, the code can be used to subset analyses to only participants who completed the retirement belief survey and the cognitive test within a year of one another. The code can also be adapted to conduct sensitivity analysis to examine how/if associations differ by amount of time allowed between the data collection dates for the two variables.

## Data Availability

Data from the dataset are publicly available through the UAS data portal while logged in on the UAS website (https://uasdata.usc.edu/index.php?r=eNpLtDKyqi62MrFSKkhMT1WyLrYyNAeyS5NyMpP1UhJLEvUSU1Ly80ASQDWJKZkpUKahoZmRknUtXDB_yBMQ). Documentation for the dataset can be found in the “UAS Cognitive Comprehensive File Data Description” document (https://uasdata.usc.edu/page/Cognitive+Comprehensive+File).

## Code availability

Code to generate all figures and tables, and example code for merging with other UAS files, are available at https://osf.io/9jp36/files/osfstorage.
